# Risk Factor Control in a Portuguese Vascular Risk Clinic: A 16-Month Retrospective Cohort Study

**DOI:** 10.7759/cureus.102735

**Published:** 2026-01-31

**Authors:** Cristiano Gante, Stanislav Tsisar, Ana Catarina Reis, Mario Rodrigues, Mariana Salvado de Morais, Luís R Almeida, Luis Dias

**Affiliations:** 1 Internal Medicine, Centro Hospitalar Universitário de Lisboa Central, Lisbon, PRT

**Keywords:** diabetes mellitus, dyslipidemia, high blood pressure, outpatient clinic, vascular risk factors

## Abstract

Background

Cardiovascular diseases (CVDs) are the leading cause of morbidity and mortality worldwide and in Portugal. Stroke remains one of the principal causes of death in Portugal, increasing the urgency of efficient primary and secondary preventive strategies.

Methods

A retrospective cohort study was conducted, including 229 patients followed at a specialized vascular risk clinic between January 2022 and April 2023, corresponding to 820 outpatient visits. Electronic medical records were retrospectively reviewed, and an anonymized database was created to collect demographic data, referral origin, cardiovascular risk factors, therapeutic management, and risk factor control. Recorded variables included age, sex, number of consultations, blood pressure, body mass index, lipid profile, fasting glucose, and glycated hemoglobin. Patients were classified into primary or secondary prevention groups; cardiovascular risk in primary prevention was assessed using SCORE2 and SCORE2-OP models. Current patterns of antihypertensive use and glycemic control were descriptively compared with data from an internal institutional study conducted in 2011-2012.

Results

A total of 229 patients were included (57% male), with a mean age of 70.5 years. Most referrals originated from inpatient hospitalization or other outpatient specialty clinics. Secondary prevention accounted for 76% of patients, and 96% of the cohort was classified as high or very high cardiovascular risk. Dyslipidemia (94%), hypertension (93%), and diabetes mellitus (63%) were highly prevalent, with 69% of patients presenting three or more cardiovascular risk factors.

Hypertension was present in 212 patients, most requiring combination therapy, and blood pressure control according to European Society of Cardiology targets was achieved in 76%. Compared with data from 2011 to 2012, prescribing patterns evolved with increased use of angiotensin-converting enzyme (ACE) inhibitors and calcium channel blockers, reduced use of angiotensin receptor blockers and diuretics, and the introduction of sodium-glucose cotransporter 2 inhibitors and mineralocorticoid receptor antagonists.

Diabetes mellitus was predominantly type 2 (94%), with a mean glycated hemoglobin of 6.6%, representing an improvement compared with a decade earlier. The use of newer antidiabetic agents, including SGLT2 inhibitors and glucagon-like peptide-1 (GLP-1) receptor agonists, was substantial despite prescription constraints. Dyslipidemia management relied mainly on statin-based therapies, frequently in combination with ezetimibe. Overweight and obesity were common, and target organ damage, particularly cerebrovascular disease and chronic kidney disease, was frequently observed.

Conclusions

Patients followed in a specialized vascular risk clinic had a very high burden of modifiable cardiovascular risk factors, with most being classified as at high or very high cardiovascular risk. Nevertheless, a substantial proportion achieved recommended targets for blood pressure and glycemic control, reflecting the impact of structured, specialized follow-up. These findings underscore the importance of comprehensive and regularly reassessed preventive strategies in aging populations.

## Introduction

Cardiovascular diseases (CVDs) are the leading cause of death globally, responsible for an estimated 19.8 million deaths in 2022, approximately 32% of all global mortality, with heart attacks and strokes accounting for 85% of these deaths [1]. In Europe, CVDs account for about 42.5% of all deaths, representing the highest regional burden globally [2]. Stroke and ischemic events contribute substantially to this mortality.

In Portugal, CVD continues to be a primary cause of mortality, particularly among older adults, with stroke among the principal contributors to death. National cardiology societies and health authorities emphasize the need for strengthened risk stratification and management as part of a national cardiovascular health strategy [3].

The main behavioral risk factors, unhealthy diet, physical inactivity, tobacco use, and harmful alcohol consumption, typically lead to intermediate risk factors, including hypertension, hyperglycemia, dyslipidemia, overweight, and obesity. These factors are measurable in primary care and strongly predict future cardiovascular events [1]. In accordance with these contributors, the pathophysiology of atherosclerotic disease involves endothelial dysfunction, chronic inflammation, lipid accumulation within the arterial intima, and plaque formation. Subsequent plaque instability or rupture can precipitate ischemic events in both the heart and cerebral circulation [4].

Despite substantial advances in cardiovascular risk stratification and pharmacological management over the past decade, real‑world data describing contemporary patterns of risk factor control and their evolution in high‑risk outpatient populations remain limited. This study aimed to characterize current cardiovascular risk profiles, management strategies, and risk factor control in a specialized vascular risk clinic and to compare these findings with institutional data from a decade earlier.

## Materials and methods

Study design

This was a retrospective cohort study conducted at a specialized vascular risk outpatient clinic. Clinical data were obtained through the retrospective review of electronic medical records of patients attending the clinic between January 2022 and April 2023. During this period, a total of 229 patients were evaluated, accounting for 820 outpatient visits. A dedicated database was created for the purpose of this study to systematically record and analyze the collected variables.

Patient recruitment

All consecutive adult patients who attended the vascular risk clinic during the study period were eligible for inclusion. Patients were followed within the context of routine clinical care, either referred from primary care or other hospital specialties. No additional interventions or visits were performed for research purposes. Patients were subsequently classified into primary or secondary cardiovascular prevention groups based on the presence or absence of established cardiovascular disease, according to standard clinical definitions.

Data collection

Clinical records were reviewed retrospectively, and a structured Microsoft Excel (Redmond, WA: Microsoft Corp.) database was developed to collect anonymized patient-level data. Variables recorded included demographic characteristics (age and sex), healthcare utilization data (number of consultations per year and number of vascular risk clinic visits), and referral origin.

Cardiovascular risk factors were systematically assessed, with a specific focus on hypertension, diabetes mellitus, dyslipidemia, obesity, and smoking status. For each risk factor, information regarding targeted pharmacological therapy and adequacy of control was recorded. Blood pressure measurements obtained during clinic visits were used to assess hypertension control. Anthropometric data, including weight and body mass index (BMI), were collected when available.

Laboratory parameters were retrieved from electronic records, including low-density lipoprotein cholesterol (LDL-C), triglycerides, fasting plasma glucose, and glycated hemoglobin (HbA1c). These values were used to evaluate metabolic control according to contemporary clinical practice standards.

Patients classified under primary prevention were further stratified using cardiovascular risk estimation tools. Systematic Coronary Risk Evaluation 2 (SCORE2) was applied to patients aged 70 years or younger, while Systematic Coronary Risk Evaluation 2-Older Persons (SCORE2-OP) was used for patients aged 70 years or older [5,6]. These risk calculators are freely accessible tools, and the original validation studies will be cited accordingly.

Statistical analysis

The statistical analysis was primarily descriptive, aimed at characterizing the study population and current patterns of cardiovascular risk factor management. Continuous variables were summarized using means and standard deviations or medians and interquartile ranges, as appropriate, while categorical variables were presented as absolute numbers and percentages.

To contextualize the findings within an evolutionary perspective, selected variables were compared with data from an internal institutional study conducted between 2011 and 2012. This comparison focused on patterns of antihypertensive therapy use and on glycemic control, assessed by glycated hemoglobin (HbA1c) levels, to evaluate changes in clinical practice and risk factor management over the past decade.

## Results

A total of 229 patients were included in the study, of whom 131 (57%) were male, and 98 (43%) were female (Table 1). The mean age of the study population was 70.5 years (range: 38-103 years). Most patients were referred from inpatient hospitalization (35%) or other outpatient specialty clinics (31%), with additional referrals from primary healthcare services (22%) and the emergency department (12%).

**Table 1:** Demographic and clinical overview of the study population. This table summarizes key characteristics of the 229 patients included in the study. The cohort comprised 57% males, with a mean age of 70.5 (range: 38-103) years. Patients were classified according to cardiovascular prevention status, with 76% managed under secondary prevention due to established cardiovascular disease. Cardiovascular risk was evaluated according to contemporary risk-stratification guidelines, with 96% of patients categorized as high- or very-high risk. Values are presented as absolute numbers, percentages, or means with ranges. Data were derived from a retrospective review of electronic medical records.

Categories	Values
Total patients	229 (57% male)
Mean age	70.5 (38-103) years
Secondary prevention	76% (n=175)
High/very high CV risk	96%

The primary reasons for referral to the vascular risk clinic were major cardiovascular risk factors and established target organ damage. Most patients (n=175, 76%) were evaluated in the context of secondary prevention, with cerebrovascular events (stroke or transient ischemic attack) accounting for 108 cases and ischemic heart disease (acute myocardial infarction or angina) in 30 patients. Chronic kidney disease (CKD) and peripheral arterial disease contributed to 73 and four referrals, respectively, while multiple concomitant risk factors were the main reason for referral in 20 patients. On primary prevention grounds, diabetes was the most frequent cause (37 referrals), followed by hypertension (17 referrals) and dyslipidemia (eight referrals). The overall prevalence of risk factors is described in Table 2.

**Table 2:** Prevalence of major cardiovascular risk factors in the study population. This table presents the frequency of key cardiovascular risk factors among the 229 patients included in the study. Dyslipidemia was the most prevalent risk factor (93.9%), followed by hypertension (92.6%) and diabetes mellitus (63.3%). Additionally, 68.9% of patients had three or more concomitant risk factors, highlighting the high overall cardiovascular risk burden in this cohort. Data were collected retrospectively from electronic medical records and are expressed as percentages of the total study population. The information provides context for risk stratification and management strategies discussed in the study.

Risk factors	Values
Dyslipidemia	93.9%
Hypertension	92.6%
Diabetes mellitus	63.3%
≥3 risk factors	68.9%

Arterial hypertension

A total of 212 patients had hypertension, including five cases (2%) of secondary hypertension due to primary hyperaldosteronism. The majority of patients required combination therapy, most commonly three antihypertensive drugs. Mean systolic and diastolic blood pressures were 131.7 mmHg (range: 85-190 mmHg) and 69.5 mmHg (range: 44-100 mmHg), respectively (Table 3). According to the European Society of Cardiology (ESC) criteria, 162 patients (76%) achieved blood pressure control, whereas 98 patients (46%) met the American Heart Association definition [7]. Among the 24% of patients not achieving ESC targets, many were older than 75 years (n=25), had advanced CKD (estimated glomerular filtration rate {eGFR} <30 mL/min/1.73 m^2^), or demonstrated intolerance to antihypertensive therapy.

**Table 3:** Hypertension characteristics and management in the study population. This table summarizes the prevalence, control, and pharmacological management of hypertension among the 212 patients identified with the condition. Essential hypertension accounted for 97.6% of cases. Mean systolic and diastolic blood pressures were 131.7 mmHg and 69.5 mmHg, respectively. Blood pressure control according to the European Society of Cardiology (ESC) targets was achieved in 76% of patients. Combination therapy with three or more antihypertensive drugs was required in 55% of cases. Data were obtained retrospectively from clinic records, and values are presented as percentages or mean blood pressure measurements, providing insight into treatment intensity and effectiveness in a high-risk outpatient population. SBP: systolic blood pressure; DBP: diastolic blood pressure

Parameters	Values
Essential hypertension	97.6%
Mean BP	SBP: 131.7 mmHg/DBP: 69.5 mmHg
BP controlled	76%
≥3 antihypertensives	55%

**Figure 1:**
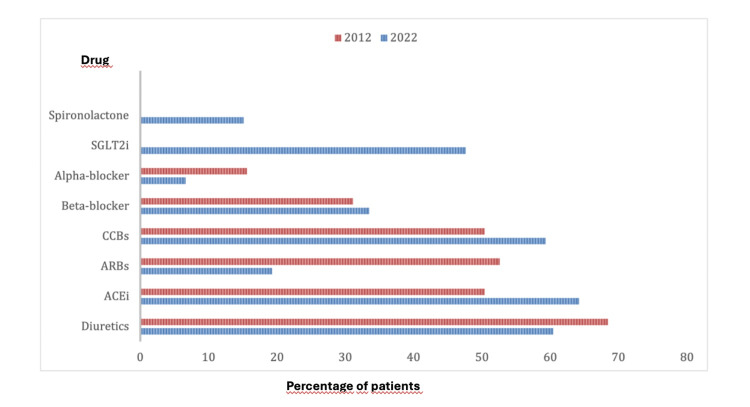
Evolution of antihypertensive therapy prescribing patterns from 2012 to 2022. This bar chart compares the proportion of patients receiving different classes of antihypertensive medications in 2012 (first bar for each drug) and 2022 (second bar for each drug) among the study population. The y-axis lists the drug classes, including ACE inhibitors, diuretics, calcium channel blockers, angiotensin receptor blockers (ARBs), alpha-blockers, beta-blockers, SGLT2 inhibitors, and spironolactone. The x-axis represents the percentage of patients receiving each therapy. Results show an increase in ACE inhibitors (50% → 64%) and calcium channel blockers (50% → 59%), a decrease in ARBs (53% → 19%) and alpha-blockers (16% → 7%), a slight increase in beta-blockers (31% → 34%), and a reduction in diuretics (68% → 60%). Notably, newer agents such as SGLT2 inhibitors and spironolactone were introduced in 2022, used in 48% and 15% of patients, respectively. Data were collected retrospectively from electronic medical records, and the chart provides a descriptive comparison of prescribing trends over the past decade.

Comparing 2012 and 2022 prescribing patterns (Figure 1), ACE inhibitors increased from 50% to 64%, diuretics decreased from 68% to 60%, calcium channel blockers increased from 50% to 59%, angiotensin receptor blockers (ARBs) decreased from 53% to 19%, alpha-blockers declined from 16% to 7%, and beta-blocker use remained stable (31-34%). Notably, SGLT2 inhibitors and spironolactone were introduced more recently, reaching 48% and 15% of patients, respectively, by 2022.

Diabetes mellitus

Diabetes was present in 145 patients (63%), 94% of whom had type 2 diabetes (Table 2). Most patients had comorbid conditions as follows: 130 with hypertension, 123 with dyslipidemia, and 112 were overweight. Thirty-eight patients were over 80 years, limiting intensive glycemic control due to hypoglycemia risk. The mean HbA1c was 6.6%, improved from 7.4% a decade earlier. Eleven patients (8%) had HbA1c between 8% and 9%, with only four under 65 years of age. Regarding therapy, 48 patients (33%) were on insulin (Table 4). Among oral agents, metformin was used in 91%, dapagliflozin in 47%, DPP-4 inhibitors in 43%, sulfonylureas in 32%, and GLP-1 receptor agonists in 29%, reflecting expanding use of newer agents despite prescription restrictions in Portugal.

**Table 4:** Diabetes mellitus characteristics and management in the study population. This table presents the prevalence, glycemic control, and pharmacological treatment among patients with diabetes mellitus (n=145). Type 2 diabetes accounted for 94% of cases. Mean glycated hemoglobin (HbA1c) was 6.6%, with 65% of patients achieving HbA1c ≤7%, reflecting adequate glycemic control in most cases. Insulin therapy was used by 33% of patients, while metformin was the most frequently prescribed oral agent (91%). Data were obtained from a retrospective review of electronic medical records and are expressed as percentages or mean HbA1c values. These findings provide insight into diabetes management strategies in a high-risk cardiovascular population.

Parameters	Values
Type 2 diabetes	94%
Mean HbA1c	6.6%
HbA1c ≤7%	65%
Insulin therapy	33%
Metformin use	91%

Dyslipidemia

Dyslipidemia was present in 215 patients (94%), with 45 (21%) exhibiting mixed dyslipidemia with hypertriglyceridemia. Statin monotherapy was used in 107 patients (47%), predominantly rosuvastatin (63%) and atorvastatin (36%). Combination therapy with statin and ezetimibe was used in 87 patients (38%), triple therapy including fenofibrate in nine patients (4%), and statin plus fenofibrate in four patients (2%). Three patients (1%) were treated with ezetimibe alone. Five patients (2%) achieved targets through lifestyle alone or received bempedoic acid (0.4%) or PCSK9 inhibitors (0.8%) due to intolerance to oral agents.

Obesity and tobacco use

Overweight and obesity were common as follows: 72 patients (31%) were overweight (BMI: 25-29.9 kg/m^2^), 41 patients (18%) had class I obesity (BMI: 30-34.9 kg/m^2^), 21 patients (9%) had class II obesity (BMI: 35-39.9 kg/m^2^), and three patients (1%) had class III obesity (BMI: ≥40 kg/m^2^). Seventeen patients (7%) were current smokers, and 65 patients (28%) were former smokers. The mean smoking history was 45.9 pack-years.

Target organ damage

Target organ damage was frequent, influencing treatment strategies. Cerebrovascular disease affected 108 patients (47%), with 87% strokes and 13% transient ischemic attack (TIA). CKD was present in 73 patients (32%), including 30 patients (13%) with stage ≥3a according to the Kidney Disease: Improving Global Outcomes (KDIGO). Cardiac disease occurred in 30 patients (13%), with 77% of whom had experienced an acute myocardial infarction and 23% reporting angina.

## Discussion

This cohort demonstrates a high burden of multiple cardiovascular risk factors, consistent with epidemiological data showing that nearly all patients with hypertension and diabetes present with concomitant risk factors, such as dyslipidemia, obesity, and hypertension [8,9]. The advanced mean age of 70.5 years underscores the importance of frailty, multimorbidity, and age‑related physiological changes in influencing treatment goals and tolerability.

Hypertension remains a key modifiable risk factor, with 76% of patients achieving ESC‑recommended targets. Nonetheless, individualized blood pressure goals are critical in older adults and frail patients to balance cardiovascular protection with the risk of hypotension and adverse events [10]. Changes in antihypertensive prescribing over the past decade, particularly the increased use of ACE inhibitors, CCBs, SGLT2 inhibitors, and spironolactone, reflect evolving guideline recommendations and emerging evidence for cardiovascular and renal protection [11].

Diabetes management shows improved glycemic control over the last decade (mean HbA1c 6.6%), despite the challenges of comorbidities, advanced age, and CKD. The increasing use of SGLT2 inhibitors and GLP‑1 receptor agonists highlights their role in high‑risk populations with multiple cardiometabolic comorbidities [12].

Dyslipidemia remains prevalent and challenging, with only a minority achieving low-density lipoprotein (LDL) targets due to older age, comorbidities, statin intolerance, and complex pharmacotherapy needs. The recent introduction of PCSK9 inhibitors and bempedoic acid offers additional options for high‑risk patients but remains limited by cost and regulatory constraints [13]. Obesity and overweight were highly prevalent, consistent with national trends, and contribute to multimorbidity and cardiovascular risk [14]. Tobacco exposure, although less common, remains relevant, particularly in those with long smoking histories [15].

Target organ damage, including cerebrovascular, renal, and cardiac disease, was frequent and necessitated ongoing adjustments in therapy, emphasizing the dynamic nature of risk management in older, high‑risk populations [8,9]. CKD, in particular, requires careful consideration of pharmacological interventions, as renal impairment alters drug metabolism and increases susceptibility to adverse effects [6]. Overall, these findings underscore the importance of patient‑centered, individualized care in high‑risk populations, balancing guideline‑based targets with age, frailty, comorbidities, and functional status to optimize cardiovascular outcomes and safety [10,13].

Limitations

Despite its clinical relevance, this study has several limitations. First, as a retrospective cohort, it relies on existing clinical records, which may lack complete data on patient adherence, frailty indices, functional status, and longitudinal outcomes beyond clinic visits. Second, the study was conducted in a specialized tertiary outpatient clinic, which may limit the generalizability of findings to broader primary care populations or to healthcare systems with different referral patterns. Third, although risk factor control was quantified, this design cannot establish causality between risk factor modification and clinical outcomes, such as cardiovascular events or mortality. Finally, we did not systematically assess patient-centered outcomes such as quality of life, which are increasingly recognized as important determinants of treatment tolerability and benefit in older adults.

## Conclusions

This cohort demonstrates a high prevalence of multiple modifiable cardiovascular risk factors, underscoring the complexity of managing high-risk, aging populations in Portugal. Optimizing risk factor control requires continuous monitoring, individualized medication adjustments, patient education, and lifestyle interventions. Long-term follow-up in a specialized vascular risk clinic offers the advantage of maintaining patients' engagement with hospital care, ensuring adherence to pharmacotherapy, and achieving reasonable control of blood pressure, glycemia, and lipids despite challenges such as advanced age, multimorbidity, and treatment intolerance. Strengthening preventive strategies and implementing guideline-driven, patient-centered care remain essential to reduce acute cardiovascular events and their associated burden.
